# Application of a simple measuring method to evaluate the fecal microflora of dairy cows in the summer season

**DOI:** 10.5455/javar.2021.h516

**Published:** 2021-06-25

**Authors:** Miki Amimoto, Yoshimitsu Ouchi, Miki Okita, Takashi Hirota, Yoshimi Imura, Takashi Bungo

**Affiliations:** 1 Laboratory of Animal Behavior and Physiology, Graduate School of Integrated Science for Life, Hiroshima University, Higashi-Hiroshima, Japan; 2 Faculty of Agriculture, University of the Ryukyus, Japan; † These authors contributed equally to this work.

**Keywords:** *Lactobacillus*, *Escherichia coli*, heat stress, feces, cows

## Abstract

**Objective:**

The effect of seasonality needs to be considered in designing future studies because global warming has caused a rise in ambient temperatures. The objective of the present study is to investigate the effect of high ambient temperatures on fecal score and fecal microflora in dairy cows during summer.

**Materials and Methods:**

During the 7 days before the sampling of feces, the daily mean temperatures were 19.9°C in early summer and more than 27.5°C in late summer. Fecal samples were collected from the rectum of cows and the fecal score was evaluated on a 4-point scale. The equalized samples were used to extract the genomic deoxyribonucleic acid (DNA) of the bacteria (*Escherichia coli*, *Salmonella*,* Lactobacillus*, and *Bifidobacterium*).

**Results:**

There was no significant difference in fecal scores between the sampling times in early and late summer. In the populations of the bacteria, there was no significant difference between sampling days in the DNA level of *Salmonella*, and *E. coli* in late summer increased to more than three times the level in early summer. However, both levels of *Lactobacillus* and *Bifidobacterium* in early summer significantly decreased after 2 months.

**Conclusion:**

These data suggest that the increase in temperature in late summer may adversely affect the populations of bacteria in the intestinal environment of dairy cows. In addition, the method used in the present study was sufficient to evaluate the changes in internal and external environmental conditions of dairy cattle.

## Introduction

In the last few decades, the consolidation of intestinal environments, such as stable microflora in the gastrointestinal tract, has been recognized as one of the critical factor for animal health and well-being. The function of the microflora partially depends on the microbial community structure [1]. A stable microbial composition plays a pivotal role in digestion and absorption [2,3], but it also competes with pathogenic bacteria in the gastrointestinal tract of animals [4]. It is known that nutritional and environmental changes can induce an imbalance in the microflora composition [5-7].

On a farm, livestock face various environmental changes, which become stressors to livestock. These stressors can affect the established protective microorganisms in animals [1,8]. The proliferation of *Lactobacillus* in the gastrointestinal tract can reduce the count of pathogenic microorganisms [9] but during a state of stress, *Lactobacillus* tends to decrease and *Escherichia coli* (opportunistic pathogens) tends to increase [10]. There is comparatively less research on the gastrointestinal or fecal microflora in adult cattle than in monogastric animals (e.g., chickens and pigs). In particular, the effect of seasonality (main effect is ambient temperature) needs to be considered in designing future studies because global warming has caused a rise in ambient temperatures.

The objective of the present study is to analyze fecal microflora using a simple measuring method to detect the effect of ambient temperatures on the intestinal environment in dairy cows.

## Materials and Methods

The animals were handled in accordance with the regulations of the Animal Experiment Committee of Hiroshima University (authorization No. E16-1) and Law No. 105 and Notification No. 6 of the Japanese government.

A total of 14 Holstein Friesian cows, which did not display any illnesses during the sample collections, were used in this investigation. The investigation was carried out in a free stall barn with an automatic milking system, Astronaut A3 next (Lely, Drachten, The Netherlands), and a roughage feeding control system (Insentec BV, Drachten, The Netherlands) at the Saijo Experimental Farm Station of Hiroshima University. In June (early summer) and August (the hottest period of summer), fecal samples (40–50 gm) were collected from the rectum of cows from 1300 h to 1500 h on the sampling days. Ambient temperatures during the week just before each sampling (June and August) are shown in Table 1. Each fresh sample was visually scored on a 4-point scale ranging from 1 to 4 (1 = runny, 2 = loose, 3 = soft, and 4 = dry) [11]. After each sample was kneaded and equalized, they were stored on wet ice and shipped to the laboratory for microflora analysis. The fecal samples were stored at –80°C prior to deoxyribonucleic acid (DNA) extraction.

Each sample was put into a sterile tube with phosphate buffer saline (PBS), and homogenized by vortexing. Clastics were abated by centrifugation at 2,000 *g*, and the supernatant fraction was collected and centrifuged at 2,000 *g*. The resulting pellet was washed twice with PBS, and re-suspended in 0.5 M ethylenediamine tetraacetic acid. The genomic DNA of the bacteria was extracted according to the standard phenol chloroform method [12]. The concentration of DNA was determined spectrophotometrically (NanoDrop ND-2000c; Thermo Scientific, Inc.). Primers for *E. coli*,* Salmonella*,* Lactobacillus*, and *Bifidobacterium* were designed according to the previous reports [13-17]. The primers used for real-time polymerase chain reaction (PCR) are shown in Table 2. Universal [16S rRNA (ribosomal ribonucleic acid)] primer for all already known bacteria was used for detecting the total microflora population.

**Table 1.** Daily mean, maximum, and minimum temperatures (°C) during the week just before sampling.

	Mean	Maximum	Minimum
	(Range)	(Range)	(Range)
June	19.9	25.6	14.1
	(18.1–21.3)	(19.6–28.4)	(11.4–17.9)
August	27.5	33.4	22.1
	(25.7–28.9)	(30.9–35.3)	(18.8–23.2)

The relative quantitation of target bacteria in fecal samples was determined according to the methods of Suzuki et al. [18]. The relative quantitation is normalized to be the numbers of target DNA copies to those of the universal gene using a simplification of the comparative threshold cycle (∆∆Ct) method. The Ct value, a critical threshold cycle, was defined as the first cycle and was inversely proportional to the logarithm of the initial number of template molecules. The ∆Ct value was calculated for the sample by subtracting the Ct value of 16S rRNA from the Ct value of the target gene. The fold difference (*N*) in the number of the target specific gene copies relative to the number of universal gene copies was calculated as follows:

*N* = 2^*∆*Ct^ = 2^(Ct target DNA – Ct universal DNA)^

The data were analyzed using the commercially available package StatView (Version 5, SAS Institute, Cary, 1998). For comparisons between the relative amounts of bacteria in June and August, all data were evaluated using the Wilcoxon signed-rank test. The level of significance was set at *p* < 0.05 and at *p* < 0.1 for a trend.

## Results and Discussion

Table 1 shows that daily mean of ambient temperatures during the week just before each sampling day. In June, there was 1 day with a maximum temperature of more than 28°C, but the mean maximum temperature was 25.6°C and the daily mean temperature was 19.9°C. In August, the daily mean temperature was more than 27.5°C during the 7 days before sampling.

**Table 2.** Microbial PCR primer sequences

Primer	Forward (5’ → 3’)	Reverse (5’ → 3’)
Universal	CGTGCCAGCCGCGGTAATACG	GGGTTGCGCTCGTTGCGGGACTTAACCCAACAT
*E. coli*	GACCTCGGTTTAGTTCACAGA	CACACGCTGACGCTGACCA
*Salmonella*	CGGGCCTCTTGCCATCAGGTG	CACATCCGACTTGACAGACCG
*Lactobacillus*	CATCCAGTGCAAACCTAAGAG	GATCCGGTGCAAACCTAAGAG
*Bifidobacterium*	GGGTGGTAATGCCGGATG	CCACCGTTACACCGGGAA

**Figure 1.**
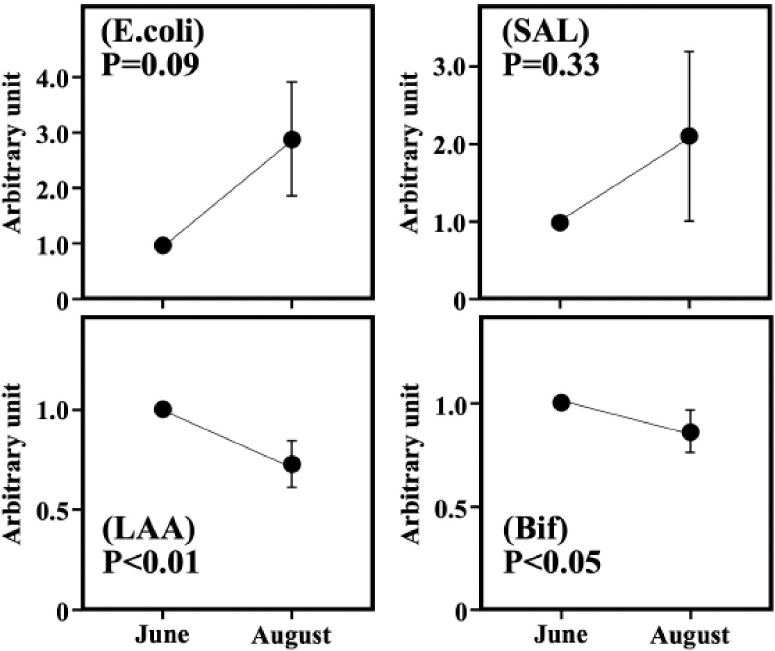
The effect of heat stress on the population of *Escherichia coli* (E. coli), *Salmonella* (SAL), *Lactobacillus* (LAA) and *Bifidobacterium* (Bif) species in fecal contents expressed in arbitrary units. Values (relative value of each cow to one in June) are means with their standard errors represented by vertical bars.

The fecal scores were 2.88 in June and 2.82 in August (data not shown). There was no significant difference between sampling times in early and late summer (*p* > 0.1).

The effect of heat stress on the populations of bacteria in the fecal contents is shown in Figure 1. Although there was no significant difference between June and August in the DNA level of *Salmonella *(*p* = 0.33), *E. coli* in August increased to more than three times the level in June (*p* < 0.1). On the contrary, both levels of *Lactobacillus* and *Bifidobacterium* in June significantly decreased after 2 months (*p* < 0.01, *p* < 0.05, respectively).

The present results showed that there was no significant difference in fecal score between the sampling times in early and late summer. In the populations of bacteria, there was no significant difference between sampling days in the DNA level of *Salmonella*, and* E. coli* in late summer increased to be more than three times the level in early summer. However, both levels of *Lactobacillus* and *Bifidobacterium* in early summer significantly decreased after 2 months. These data suggest that the increase in temperature in late summer may increase the populations of unfavorable bacteria and decrease those of beneficial bacteria in the intestinal environment of cows.

It is well known that an ambient temperature of 5°C to 25°C, called the thermoneutral zone, is comfortable for dairy cows, whereas they are not able to cool themselves adequately and become heat stressed when the ambient temperature exceeds 26°C [19]. Although there were no data on behavioral observation, we found that all cows displayed a sign of heat stress (panting) in August. Therefore, it seemed that they were in a state of severe heat stress at the time of sampling in August but not in June.

The proper state of microflora plays a significant protective and nutritional role in the digestive tract [20]. Exposure to environmental stressors can significantly affect the microbiota community structure in animals [1,10], which consequently causes an imbalance in microflora. The pathogenic bacteria and imbalanced microflora in the digestive tract could produce toxins that decrease nutritional and immune abilities, leading to disease. It has been shown that diarrhea in calves occurs through this mechanism [5,21,22]. Although our result for fecal score showed that no cows had symptoms of diarrhea, their digestion and absorption ability might decrease through an imbalance in microflora induced by heat stress. Evidence suggests that an imbalance in microflora affects not only digestion and absorption but also the stress response and behavior of the central integrative systems of animals [23]. Collectively, the proper state of intestinal microflora is important in contributing to the health and well-being of livestock.

An increase in the populations of unfavorable bacteria may contaminate the rearing environment in cows. The environmental pathogen *E. coli* can be found in the bedding materials, floors, and manure in a cow’s environment [24]. In addition, *E. coli* is a primary pathogen in infectious diseases in the bovine udder [25-27], and *Salmonella* is also an opportunistic bacterium that emerges when a cow’s health is compromised [28,29]. Thus, the present results imply that heat stress in late summer increases the risk of infectious diseases such as mastitis as determined by an increase in unfavorable bacteria in their feces. Because the microbial communities alter across the intestinal segments in dairy cattle [30,31], the bacterial community in feces may not completely reflect those in other sites of the digestive tract. However, the method used in the present study was sufficient to evaluate the changes in internal and external environmental conditions of dairy cattle.

## Conclusion

The present study demonstrates that heat stress in the summer may increase the populations of unfavorable bacteria and decrease those of beneficial bacteria in the intestinal environment of cows. In addition, the possibility of infection could increase through contamination by excreta pathogens such as *E. coli* in the rearing environment of cows. Because the present study evaluated the transition of both beneficial and unfavorable bacteria in feces, further research should appraise the seasonal changes in diversity or composition of microflora in each intestinal segment of dairy cattle.

## List of Abbreviations

gm: grams; h: hour; DNA: deoxyribonucleic acid; RNA: ribonucleic acid; *E. coli* = *Escherichia coli.*
